# Angiogenesis and targeted therapy in the tumour microenvironment: From basic to clinical practice

**DOI:** 10.1002/ctm2.70313

**Published:** 2025-04-23

**Authors:** Shuaixi Yang, Yingshuai Fang, Yangcheng Ma, Fuqi Wang, Yuhang Wang, Jiachi Jia, Yabing Yang, Weipeng Sun, Quanbo Zhou, Zhen Li

**Affiliations:** ^1^ Department of Colorectal Surgery The First Affiliated Hospital of Zhengzhou University Zhengzhou China; ^2^ The First Clinical School of Medicine Zhengzhou University Zhengzhou China; ^3^ Department of Orthopedics The First Affiliated Hospital of Zhengzhou University Zhengzhou China

**Keywords:** angiogenesis, antiangiogenic therapy, cancer, EndMT, endothelial cells

## Abstract

Angiogenesis, as a core marker of cancer survival and growth, is integral to the processes of tumour growth, invasion and metastasis. In recent years, targeted angiogenesis treatment strategies have gradually become an important direction in cancer treatment. Single‐cell sequencing technology can provide new insights into targeted angiogenesis by providing a deeper understanding of the heterogeneity of tumour endothelial cells and exploring the interactions between endothelial cells and surrounding cells in the tumour microenvironment. Here, we systematically review the research progress in endothelial cell pathophysiology and its endothelial‒mesenchymal transition and illustrate the heterogeneity of endothelial cells from a single‐cell perspective. Finally, we examine the contributions of different cell types within the tumour microenvironment in relation to tumour angiogenesis, as well as the latest progress and strategies in targeted angiogenesis therapy, hoping to provide useful insights into the clinical application of antiangiogenic treatment. Furthermore, a summary of the present progress in the development of potential angiogenesis inhibitors and the ongoing clinical trials for combination therapies is provided.

**Key points:**

- Angiogenesis plays a key role in tumour progression, invasion and metastasis, so strategies targeting angiogenesis are gradually becoming an important direction in cancer therapy.
- Interactions between endothelial cells and stromal cells and immune cells in the tumour microenvironment are significant in angiogenesis.
- The application of antiangiogenic immunotherapy and nanotechnology in antiangiogenic therapy provides a vital strategy for prolonging the survival of cancer patients.

## Graphical abstract

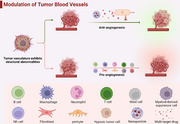

## 1 INTRODUCTION

In the past few years, there has been a consistent annual decline in the mortality rate among cancer patients, but tumour metastasis remains a major challenge. This not only complicates the treatment of malignant tumours but also becomes an important cause of recurrence after surgical removal of tumours. The tumour vasculature acts as a critical regulator of metastasis, significantly affecting tumour progression and providing pathways for dissemination.^1^ Since Folkman's 1971 theory that ‘tumours depend on angiogenesis and that angiogenesis inhibitors can treat cancer’, endothelial cells (ECs) have received heightened interest within the domain of cancer treatment.^2^ A clinical trial in 2003 demonstrated that the combination of chemotherapy and bevacizumab could extend patients' (with colorectal cancer) lives, validating the effectiveness of antiangiogenic therapies (AATs) in cancer treatment. AAT has since been identified as a promising anticancer therapeutic approach by studies of several proangiogenic and antiangiogenic molecules within the tumour microenvironment (TME).^3^ Consequently, there has been a growing number of antiangiogenic agents that have received FDA approval for clinical use. Among these, small‐molecule tyrosine kinase inhibitors and monoclonal antibodies have emerged as standard therapeutic options in this domain. However, challenges remain regarding the efficacy of AAT, as well as the lack of effective biomarkers. These issues may be associated with the adoption of alternative angiogenic modes by tumours and drug resistance mechanisms. This has led to research into other ways to enhance therapy results, such as combination therapies and the application of nanodrug delivery systems.

In this paper, we summarise the advancements in the pathophysiology of ECs and endothelial‒mesenchymal transition (EndMT),^4^, ^5^ highlighting the heterogeneity of ECs from a single‐cell perspective. We also emphasise the contributions of various cell types in the TME to tumour angiogenesis. In conclusion, we present a comprehensive summary of the latest developments and tactics in targeted angiogenesis therapy, aiming to offer valuable insights for the clinical application of antiangiogenic treatments.

## 2 ADVANCES IN UNDERSTANDING THE PATHOPHYSIOLOGY OF ECS

### 2.1 Origins and tumour vasculature

The endothelium of arteries, veins and capillaries in humans is made up of flat, single‐layered cells called ECs that are mostly found on the inner walls of blood vessels and come into close touch with the blood's constituent cells and components.^6^ It is now widely accepted that ECs originate from the mesoderm during the embryonic period.^7^ Research has indicated that the transcription factor families FOX and ETS play important roles in the specialisation and establishment of ECs.^8^ Under normal conditions, ECs are typically in a quiescent state, with a relatively low proliferation rate. Under physiological conditions, ECs proliferate approximately once every 150 days, contributing to the blood vessels' stability and integrity.^9^

However, during tumourigenesis, the proliferation rate of ECs significantly increases. Owing to the hypoxic conditions in the tissue, tumour cells (TCs) secrete various angiogenic factors, including vascular endothelial growth factor (VEGF), which cause ECs in the adjacent normal tissue to proliferate and migrate. Thus, new blood vessels are formed. TCs grow and proliferate when oxygen and nutrients are delivered by blood arteries.^10^ In contrast to normal blood vessels, tumour vasculature exhibits characteristics such as tortuosity, intertwining, dilation and disorganisation. They also have loose pericyte covering and leaky neovasculature, which raises vascular permeability and, in turn, interstitial pressure. High interstitial pressure further causes vessel collapse and reduced TC perfusion, creating a hypoxic and acidic tumour immune microenvironment. Vascular abnormalities and compromised perfusion hinder the infiltration of immune cells and antitumour agents from the circulatory system into the tumour, suppressing antitumour activity.^11^

**FIGURE 1:**
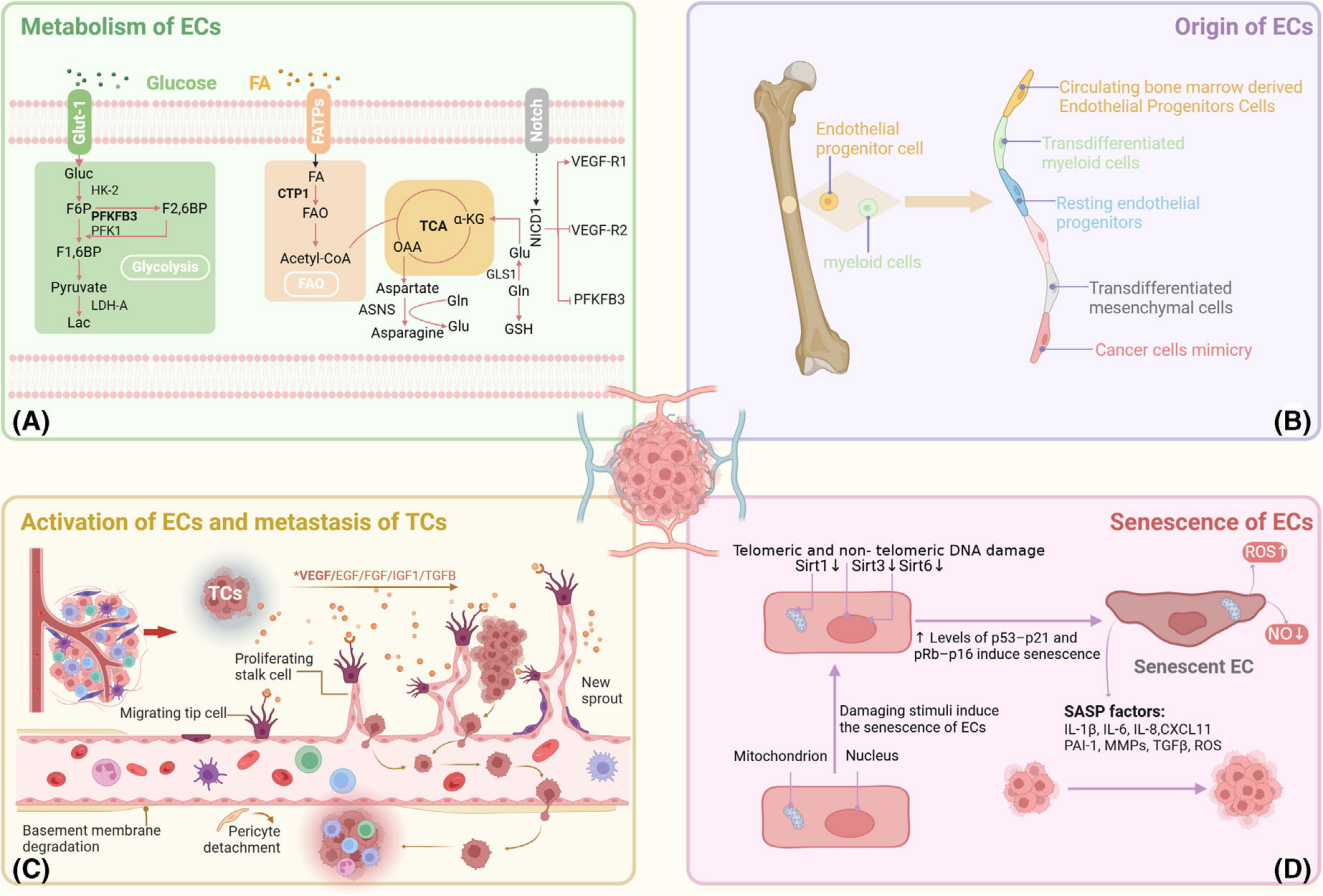
Metabolism, origin, activation and senescence of endothelial cells (ECs), along with the metastasis of tumour cells (TCs). (a) Glycolysis and fatty acid oxidation (FAO) in ECs, with PFKFB3 as the key enzyme for glycolysis and CPT1a as the pivotal enzyme for FAO. (b) Diverse origins of tumour endothelial cells (TECs). (c) Sprouting angiogenesis and tumour metastasis. (d) Injury‐induced ECs senescence, resulting in telomere dysfunction in ECs, damage to non‐telomeric DNA, and alterations in energy‐sensitive pathways; MMPs, matrix metalloproteinases; TGF‐β, transforming growth factor Beta.

Typically, tumour angiogenesis manifests through various patterns. Sprouting angiogenesis, which is brought on by tip cell migration and proliferation, is the most characteristic process of both normal and pathological angiogenesis, resulting in new branches forming within existing blood vessels. An existing vessel is divided into two vessels during intussusceptive angiogenesis (IA), which are used by TCs to expand and invade. Endothelial progenitor cells (EPCs) derived from bone marrow, as well as cancer stem‐like cells, can also differentiate into ECs, forming new vasculature. Additionally, in order to enter the tumour, TCs can approach and take over preexisting blood arteries. Ultimately, TCs can specify ‘avascular’ channels to facilitate the transportation of oxygen and essential nutrients. Various patterns of angiogenesis may be associated with tumour resistance to therapy. Tumour endothelial cells (TECs) have unique metabolic pathways increased fructose metabolism activation, fatty acid oxidation and glycolysis, and the origin of TECs can differ from that of normal ECs^12,^ ^13^ (Figure 1).

### 2.2 Senescence

#### 2.2.1 Mechanisms of EC senescence

One of the main mechanisms behind vascular disorders such as atherosclerosis and vascular ageing is EC senescence. Current studies indicate that endothelial senescence is a complex, multifactorial process involving mitochondrial function, the immune response, miRNAs, oxidative stress and the external environment.^14^ These stimuli can activate tumour suppressor pathways, including the pRb‐p16 and p53‐p21 pathways, leading to endothelial proliferation arrest and senescence. Mitochondrial sirtuins (SIRTs), particularly SIRT3, 4 and 5, play crucial roles in maintaining endothelial homeostasis. High‐glucose conditions and CR6‐interacting factor 1 deficiency can suppress SIRT expression, resulting in premature endothelial senescence.^15^, ^16^ miRNAs also play important roles in regulating SIRT expression and endothelial function, especially miRNAs associated with mTOR in the modulation of endothelial senescence.^17^, ^18^ Oxidative stress is another key factor that promotes endothelial senescence. Studies have shown that H_2_O_2_ downregulates SIRT7 via microRNA‐335‐5p, highlighting the impact of oxidative stress on endothelial senescence.^19^ Moreover, macrophages can accelerate endothelial senescence by accumulating reactive oxygen species (ROS), especially under diabetic conditions, where M1‐type macrophages exacerbate endothelial dysfunction by enhancing inflammatory responses.^20^, ^21^ Interestingly, environmental factors such as cigarette smoke extract can also promote premature senescence via stimulating the p300–p53/p21 pathway and upregulating USP7.^22^ Notably, Hwang et al. summarised the molecular mechanisms and signalling pathways affecting endothelial senescence.^23^ Therefore, gaining a comprehensive grasp of the mechanisms of EC senescence and how it affects angiogenesis is crucial for developing effective angiogenic intervention strategies.

#### 2.2.2 Duality of EC senescence on tumour progression

Endothelial senescence significantly contributes to tumour progression via modulating the TME and influencing tumour outcomes. Although increasing research has concentrated on the protumourigenic effects of senescent ECs 24, their influence varies depending on the kind of cell, the TME's condition, and the immune system's function.^24^

On the one hand, senescent ECs exhibit pronounced senescence features in various cancers, indicating their important role in cancer development.^25^ Senescent ECs promote TC proliferation, migration and invasion via releasing senescence‐associated secretory phenotype factors, such as ROS and inflammatory chemokines, while simultaneously suppressing immune cell functions.^26^, ^27^ These cells can also exacerbate inflammation in the TME by recruiting platelets.^28^ The secretion of ROS by senescent ECs is closely related to mitochondrial dysfunction.^29^, ^30^, ^31^ In breast cancer, endothelial senescence enhances TC invasiveness by promoting the secretion of CXCL11.^32^ Surprisingly, sunitinib promotes premetastatic niche (PMN) formation in metastatic breast cancer by inducing EC senescence.^33^ Moreover, senescent ECs are associated with the liver metastasis of uveal melanoma, with KLF4 identified as a driver of endothelial senescence.^34^ By releasing substances such as interleukin‐6 (IL‐6), senescent ECs also disrupt the immune surveillance role of CD8+ T cells and encourage the recruitment of myeloid‐derived suppressor cells (MDSCs), which reduces the immune system's capacity to eradicate malignancies.^35^

On the other hand, EC senescence may also exhibit antitumourigenic effects. In some cases, senescent cells inhibit TC proliferation by activating tumour suppressor pathways that suppress the cell cycle. Additionally, EPCs play crucial roles in angiogenesis. miR‐34a has been identified as a regulator of EPC senescence that inhibits angiogenesis and tumour progression by suppressing SIRT1.^36^ Recent studies indicate that the upregulation of miR‐409 in senescent EPCs results in decreased secretion of CCL5, consequently inhibiting angiogenic activity.^36^ In conclusion, the significance of EC senescence in tumour angiogenesis and cancer progression is complex and multifaceted (Figure 1).

## 3 HETEROGENEITY AND CHARACTERISTICS OF ECS IN THE TME

### 3.1 Interaction between TECs and TCs

The interaction between TCs and ECs drives tumour angiogenesis and vascular dysfunction through multiple mechanisms. Tumour angiogenesis is a multifaceted process that entails HIF‐1 activation, angiogenic agent release and remodelling of the TME. Under hypoxic conditions, TCs activate HIF‐1 to induce bFGF and VEGF expression, enhancing EC proliferation and vascular permeability. HIF‐1 also upregulates the expression of matrix metalloproteinases (MMPs), which degrade the extracellular matrix (ECM), facilitating EC migration.^5^ Furthermore, TCs modulate EC phenotypes to favour angiogenesis. For example, TCs induce ECs to express specific integrins (e.g., αvβ3 and αvβ5), which are critical for angiogenic processes.^37^

**FIGURE 2:**
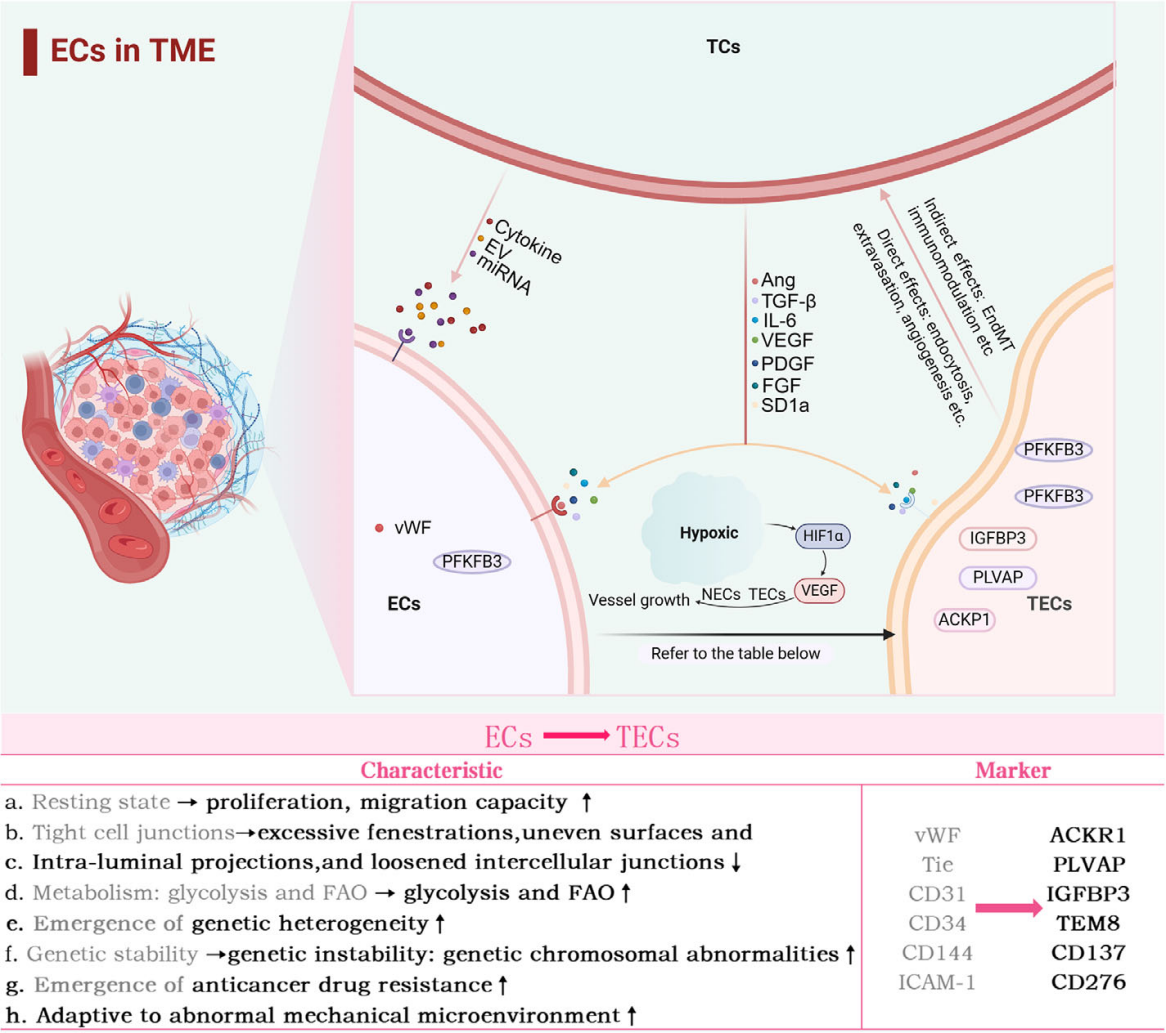
Normal endothelial cell (NEC), tumour endothelial cell (TEC) and tumour cell (TC) in the tumour microenvironment (TME). Within the TME, NECs are activated by various growth factors, chemokines or extracellular vesicles secreted by TCs, which transform into TECs. Compared with NECs, TECs exhibit specific tumour endothelial markers, greater metabolic activity, resistance to antitumour agents and adaptability to the TME. TECs directly facilitate TC proliferation and metastasis by either loosening endothelial cell (EC) junctions to allow TC infiltration and extravasation or supplying nutrients and oxygen to TCs through angiogenesis. TECs also indirectly impact TCs through immunomodulation (inhibiting effector lymphocyte infiltration), extracellular matrix (ECM) remodelling and the endothelial‒mesenchymal transition (EndMT) process; TGF‐β, transforming growth factor Beta.

Vascular dysfunction manifests as hyperpermeability, hypoxia, an acidic TME, and impaired drug delivery. Multiple vascular‐disrupting factors contribute to tumour progression. Leucine‐rich α‐2 glycoprotein 1 (LRG), a key vascular‐disrupting factor, drives vascular dysfunction, tumour metastasis and resistance to checkpoint inhibitors in melanoma.^38^, ^39^, ^40^, ^41^, ^42^ Tumour‐derived PAK4 phosphorylates VE‐cadherin, disrupting EC junctions, increasing vascular permeability and promoting TC extravasation, a process notably observed in aggressive tumours such as glioblastoma.^43^ Loss of CD93 and CX3CL1 overexpression mediate aberrant EC‒ECM adhesion, resulting in the formation of disorganised vascular networks.^44^, ^45^ Additionally, PHGDH hyperactivation in the TME reprograms serine metabolism to provide biosynthetic precursors for ECs, supporting their abnormal proliferation and fostering vascular heterogeneity.^46^, ^47^ These mechanisms collectively lead to structurally and functionally compromised neovasculature (Figure 2).

### 3.2 TECs and tumour metastasis

ECs play pivotal roles in multiple phases of tumour metastasis.^48^, ^49^, ^50^ During the invasion phase, EC promotes tumour invasion by modulating the microenvironment and activating Notch signalling via CXCL12 and transforming growth factor Beta (TGF‐β).

Vascular hyperpermeability is crucial for tumour metastasis. During extravasation, ECs interact with circulating tumour cells (CTCs) to facilitate their transmigration across the vascular wall into new tissues. This process involves adhesion and signalling between TCs and TECs. Tumour‐derived molecules such as ADAM17, miR‐27b‐3p and miR‐25‐3p compromise the integrity of the endothelial barrier by targeting ZO‐1, occludin, claudin and VE‐cadherin, thereby promoting vascular permeability.^51^, ^52^, ^53^ Recent studies have revealed that CCR2 expression and pyroptosis‐related proteins in ECs induce endothelial retraction and CTC extravasation.^54^, ^55^

During circulation, endothelial surface adhesion molecules are crucial for immune evasion and metastatic migration. One mechanism involves the unresponsive state of TECs to inflammatory signals, termed endothelial anergy.^56^ VEGF and FGF2 induce endothelial anergy by suppressing vascular cell adhesion molecule 1 (VCAM‐1) and intercellular adhesion molecule 1 (ICAM‐1) expression, impairing leukocyte extravasation as well as tumour immune surveillance.^57^ Additionally, proinflammatory signals upregulate PD‐L1 and FasL expression in ECs, reducing the efficacy of immunotherapy.^58^, ^59^ Moreover, the maturation of dendritic cells is inhibited, leading to compromised antigen presentation and a subsequent inability to activate T cells.^60^ Growing evidence suggests that antiangiogenic drugs can reverse endothelial anergy and improve immunotherapy outcomes, highlighting the need for future research on the immunotherapeutic potential of these agents.^61^, ^62^ On the other hand, tumour metastasis may potentially be impacted by adhesion molecule expression. For example, E‐selectin binds to certain TCs, facilitating CTC migration across the endothelium.^63^

During the colonisation phase, ECs support TC growth and proliferation by forming new vascular networks. Some TCs can maintain a dormant state through endothelium‐derived thrombospondin‐1 and are activated during future metastasis.^64^, ^65^

### 3.3 Heterogeneity of TECs from a single‐cell perspective

The differences in the efficacy and resistance of AATs among various patients highlight the heterogeneity of TECs. Zeng et al. provided an overview of the heterogeneity observed in human TECs as examined through single‐cell methodologies across various tumour types. In malignancies such as colorectal cancer and hepatocellular carcinoma (HCC), TECs exhibit high heterogeneity. Of particular note, the three most commonly seen TEC markers are IGFBP3, ACKR1 and PLVAP, with widespread expression across multiple cancer types.^4^ Li et al. amalgamated single‐cell RNA sequencing data from 575 patients with cancer to establish a comprehensive pan cancer EC atlas and discovered two notable subpopulations: SELE+ veins and CXCR4+ tip cells. These subpopulations may be linked to response rates to AATs and immunotherapies across different cancer types. In most cancers, the abundance of the proinflammatory SELE+ vein subpopulation decreases as the tumour progresses, whereas the CXCR4+ proangiogenic tip cell subpopulation increases.^66^ Pan et al. conducted the research that produced a detailed atlas of tumour vasculature at the single‐cell level, encompassing almost 200 000 cells across 31 cancer types, elucidating the intricacies of tumour vasculature. Pan defined the stages of angiogenesis as SI, SII and SIII, noting that poor overall survival and progression‐free survival are predicted by significant infiltration of APLN+ TipSI cells. However, treatment with bevacizumab significantly reduces their infiltration, enhancing patient responses to AAT. Moreover, APLN+ TipSI cells can induce T‐cell dysfunction, contributing to an immunosuppressive environment that promotes angiogenesis.^67^ Zheng et al. identified subpopulations of tip cells expressing insulin receptors and placental growth factor. INSR+ tip cells, which highly express vascular endothelial growth factor receptor 1/2 (VEGFR), are key drivers of angiogenesis. Zheng's work positions the insulin receptor as a novel target for AAT. The above results further emphasise the significance of EC subpopulations in immunological modulation, with important implications for immunotherapeutic strategies within the TME.^68^

**FIGURE 3:**
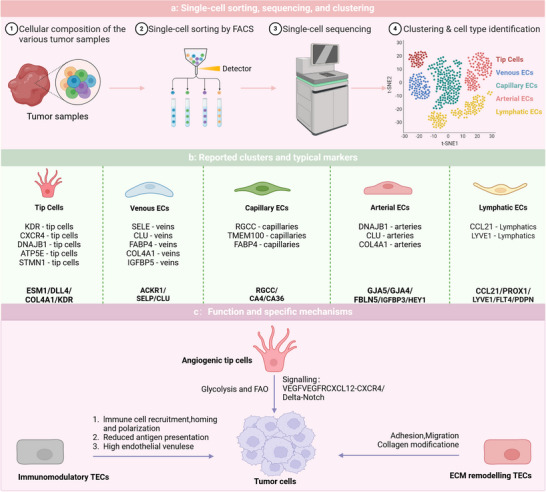
Heterogeneity of tumour endothelial cells (TECs) from a single‐cell perspective. (a) Single‐cell sequencing steps: TEC sorting, sequencing and clustering. (b) Reported clusters (middle of the diagram) and typical markers (bottom of the diagram). (c) Primary functions and specific mechanisms of TECs; TGF‐β, transforming growth factor Beta.

Despite the clear advantages of single‐cell technologies in revealing heterogeneity, challenges remain owing to the minimal sample sizes, restricted variety of cancer types examined, and the scarce presence of ECs (Figure 3).

## 4 ROLE OF ENDMT IN THE TME

**FIGURE 4:**
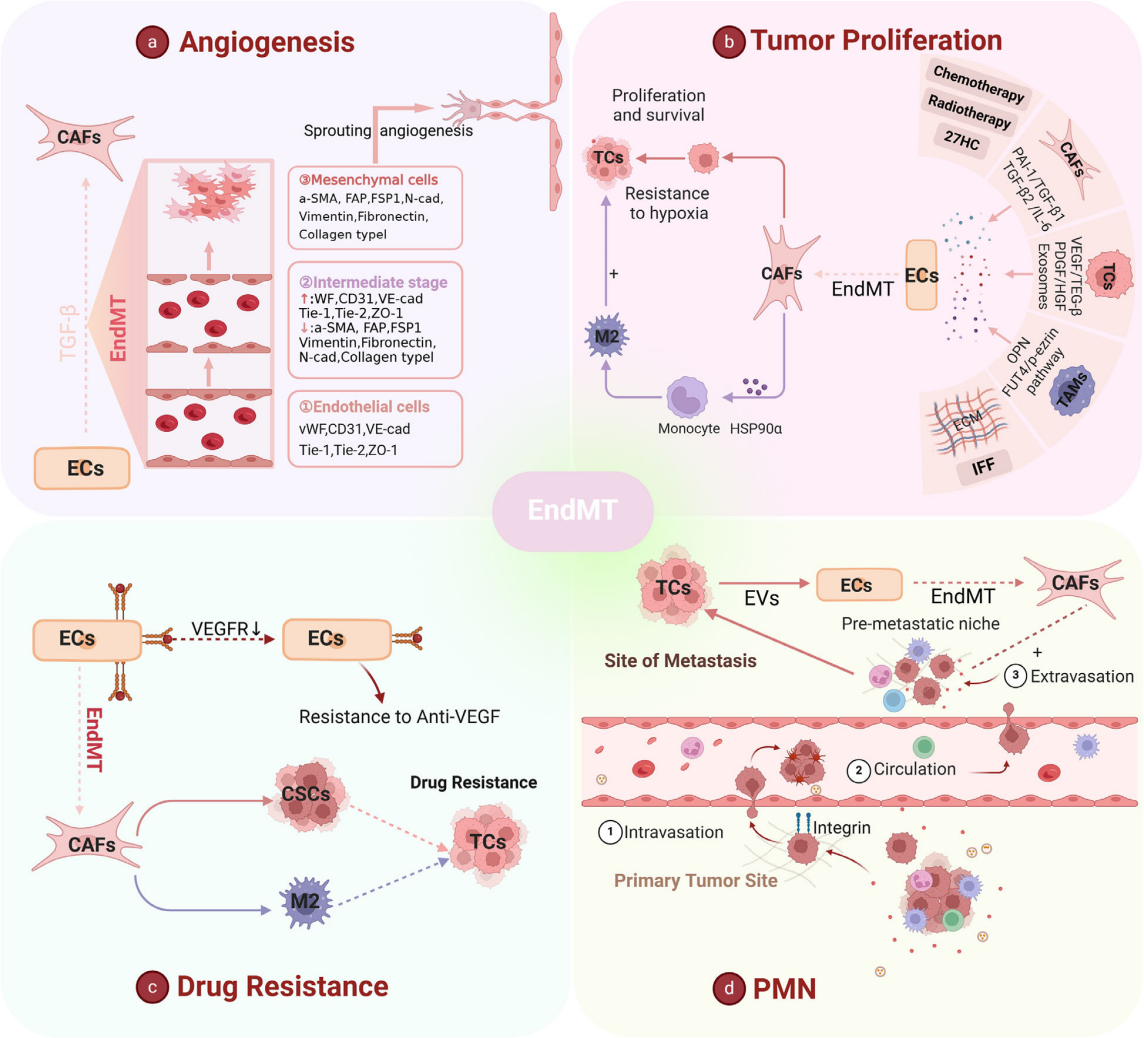
Relationships of endothelial‒mesenchymal transition (EndMT) with tumour proliferation, the premetastatic niche (PMN), tumour angiogenesis and drug resistance. (a) Tumour endothelial cells (TECs) exhibit endothelial plasticity; under the influence of TGF‐β, endothelial cells (ECs)‐specific protein markers decreased and mesenchymal protein expression markers increased. (b) Under the influence of various factors, ECs transdifferentiate into cancer‐associated fibroblasts (CAFs), which facilitate the resistance of tumour cells (TCs) to hypoxia and the evasion of immune responses. (c) EndMT participates in establishing a supportive perivascular niche for drug resistance through various mechanisms. (d) Within the PMN, TCs induce EndMT through the release of extracellular vesicles, thereby promoting TC infiltration and metastasis and enhancing angiogenesis; FGF‐2, fibroblast growth factor 2; MMPs, matrix metalloproteinases; PDGF‐C, platelet‐derived growth factor C; TGF‐β, transforming growth factor Beta.

EndMT is a biological process wherein ECs gradually acquire mesenchymal characteristics through specific signalling pathways and transcription factors. This process frequently occurs in the TME and is implicated in angiogenesis, tumour proliferation, drug resistance and PMN formation.^69^, ^70^ Therefore, comprehending EndMT in relation to tumour metastasis and recognising it to be a critical adaptation mechanism within the TME are of paramount importance (Figure 4).

### 4.1 EndMT and tumour angiogenesis

Under pathological conditions, EndMT leads to vascular abnormalities and tumour metastasis.^71^, ^72^, ^73^, ^74^ Although TECs undergoing EndMT exhibit reduced intrinsic angiogenic capacity, they promote angiogenesis via paracrine signalling and strengthen TC invasion and metastasis.^75^, ^76^, ^77^ Research has shown that neutrophil extracellular trap‐induced EndMT impairs angiogenesis and delays diabetic wound healing.^78^ Tumour necrosis factor‐alpha (TNF‐α) robustly triggers EndMT to drive angiogenesis and stromal development in pancreatic cancer.^79^, ^80^ Furthermore, EndMT markers correlate positively with microvessel density in breast cancer, suggesting a link between EndMT and poor tumour prognosis. Notably, partial EndMT that produces cells exhibiting both endothelium and mesenchymal characteristics is characterised as a transient and reversible phenomenon^81^, ^82^ (Figure 4a).

### 4.2 EndMT and tumour proliferation

EndMT enhances tumour invasiveness by promoting the mesenchymal transition of ECs. TAMs promote EndMT by releasing osteopontin (OPN), whereas low shear stress and interstitial fluid flow have been proven to upregulate EndMT as well.^77^, ^83^, ^84^ In addition, high cholesterol metabolites, as well as radiotherapy and chemotherapy, play important roles in the regulation of EndMT.^85^, ^86^, ^87^, ^88^ Notably, the induction mechanisms of EndMT include the Notch signalling pathway, TGF‐β, and other factors, as well as exosomes, and TGF‐β works in synergy with TNF‐α, IL‐1β and Notch to regulate EndMT.^75^, ^76^, ^80^, ^89^, ^90^, ^91^ EndMT is a significant source of cancer‐associated fibroblasts (CAFs) (more than 50% in gliomas and over 40% in melanomas) and CAFs differentiated from EndMT secrete HSP90α to promote the polarisation of M2 macrophages^92^, ^93^, ^94^ (Figure 4b).

### 4.3 EndMT and drug resistance

It has been found that the reversal of EndMT in lung cancer helps to mitigate chemoresistance, independent of soluble TGF‐β levels.^95^ EndMT appears to facilitate the formation of a perivascular niche that supports drug resistance in multiple ways: promoting hypoxia, drug delivery failure, disappearance of therapeutic targets and enlistment of protumour immune cells. For example, inhibiting EndMT can significantly increase glioma cells' susceptibility to temozolomide, which is undoubtedly related to drug delivery failure and hypoxia.^92^ In TECs that undergo EndMT, VEGFR2 expression is reduced, resulting in resistance of glioblastoma ECs to VEGF treatment.^74^ Moreover, EndMT promotes resistance to radiotherapy in colorectal cancer by awakening cancer stem cells and polarising M2 TAMs^86^ (Figure 4c).

### 4.4 EndMT and PMNs

PMNs have attracted much more attention in targeted cancer therapy, representing an abnormal, tumour favouring and cancer cell‐free microenvironment that is essential for the metastasis of TCs to remote organs.^96^, ^97^ EndMT in PMNs can promote the extravasation and metastasis of TCs. Extracellular vesicles released from breast cancer cells induce EndMT in liver sinusoidal ECs,^98^ indicating that TCs tend to induce EndMT at metastatic sites.^98^ Therefore, we conclude that EndMT has a specific function in the formation of PMNs. Future elucidation of the sequence of events in which EndMT occurs in tumour metastasis is anticipated to offer a novel approach to cancer therapy (Figure 4d).

## 5 CONTRIBUTIONS OF CELLS WITHIN THE TME TO ANGIOGENESIS

**FIGURE 5:**
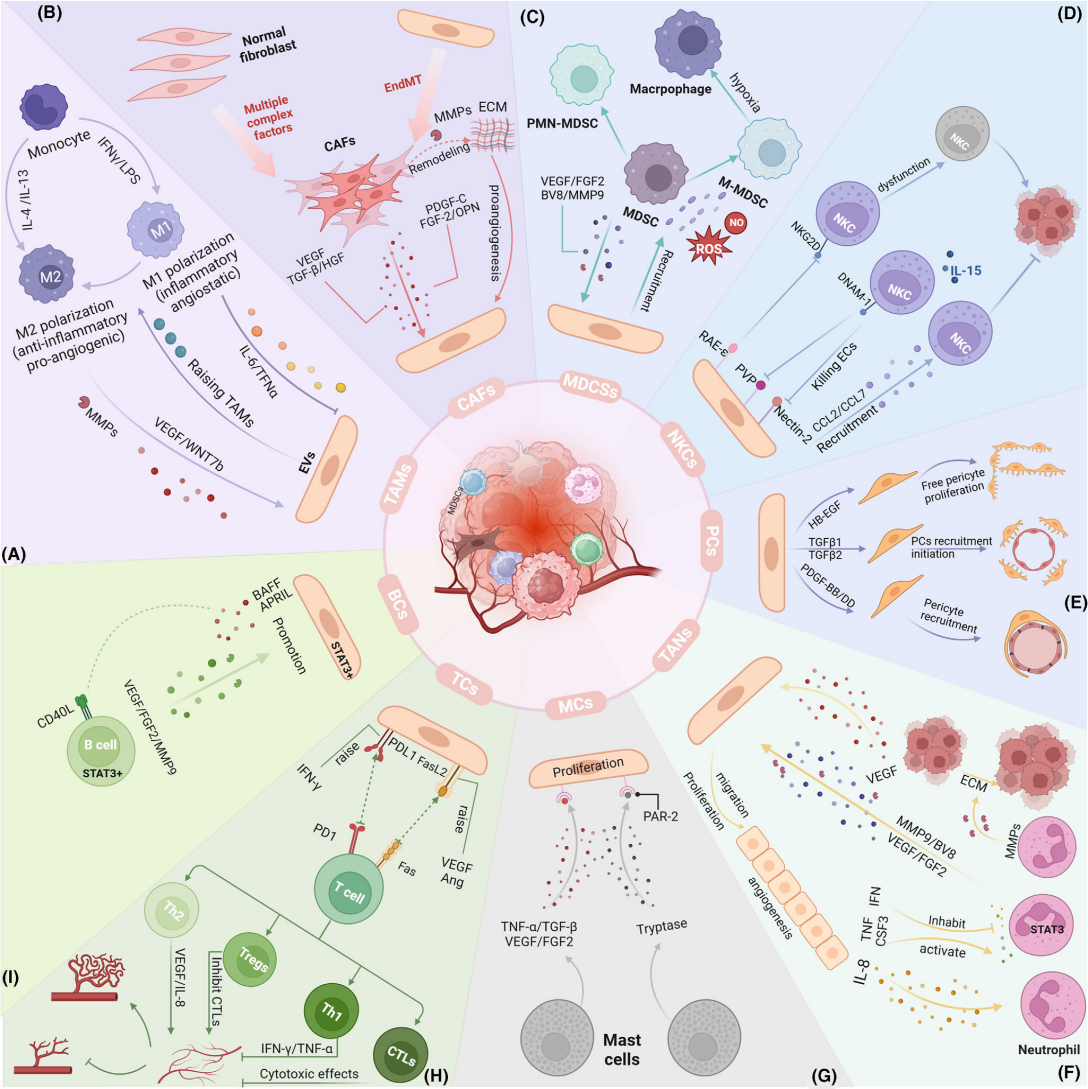
Contributions of cells within the tumour microenvironment (TME) to angiogenesis. (a) M1 macrophages secrete interleukin‐6 (IL‐6) and tumour necrosis factor‐alpha (TNF‐α), inhibiting angiogenesis, while M2 macrophages release WNT7b, accelerating tumour vascularisation. (b) Cancer‐associated fibroblasts (CAFs) support angiogenesis by secreting vascular endothelial growth factor (VEGF)‐A and PDGF‐C and inducing factors such as FGF‐2 and osteopontin (OPN). (c) Myeloid‐derived suppressor cells (MDSCs) in the TME secrete VEGF, bFGF and Bv8 to promote neovascularisation. (d) The expression of RAE‐1ε by endothelial cells (ECs) activates NKG2D, leading to dysfunction of Natural Killer cells (NK cells) and diminishing their antitumour effects. However, NK cells stimulated by IL‐15 efficiently kill tumour endothelial cells (TECs) that overexpress poliovirus receptor and Nectin‐2. (e) ECs secrete HB‐EGF, TGFβ1/2 and Platelet‐Derived Growth Factor Subunit B (PDGF‐BB)/DD to promote the proliferation and recruitment of PCs. (f) The activation and inhibition of IFN, TNF and CSF3 are related to TANs, which release VEGF‐A, FGF2 and MMP9 alongside VEGF secreted by tumour cells (TCs), collaboratively promoting angiogenesis. (g) Proteases secreted by mast cells (MCs), such as tryptase and chymase, bind to PAR2 on ECs, facilitating tumour angiogenesis and TC proliferation. (h) Th2 cells and Tregs promote neovascularisation, whereas Th1 cells and cytotoxic T lymphocytes (CTLs) impede angiogenesis and the proliferation of TCs. (i) B cells release VEGF‐A, FGF2 and MMP9, activating STAT3 within ECs and promoting their proliferation.

Over the past few decades, our view of targeting tumour vasculature has changed significantly as our understanding of its complexity and complex interactions with other cell types has increased. Among these, the interactions between different cell types in the TME and ECs have been well studied for their effects on angiogenesis. The function of these cells in tumour angiogenesis is discussed below (Figure 5).

### 5.1 TAMs

Macrophages exhibit significant plasticity in regulating tumour angiogenesis. TAMs are currently believed to alter the transcriptional programs in reaction to cues from TME, forming a continuous spectrum, with the extremes being the M1 and M2 phenotypes.^99^ M1 TAMs suppress tumour angiogenesis, whereas M2 TAMs enhance angiogenesis.^100^ Cytokines such as IL‐4 and IL‐13 can polarise macrophages towards the M2 phenotype, leading to the secretion of proangiogenic mediators, including VEGF, TGF‐β and MMPs.^101^, ^102^, ^103^ In contrast, M1 TAMs induced by lipopolysaccharides and interferon‐γ (IFN‐γ) suppress angiogenesis by releasing proinflammatory factors, including IL‐6 and TNF‐α. Conversely, the TEM represents a unique subset of macrophages. Tie‐2 on the surface of the TME promotes tumour angiogenesis by binding with ANGPT2. A recently identified subset of proangiogenic macrophages (PraM) is widely distributed across multiple tissues and promotes angiogenesis through the expression of high levels of angiogenesis‐promoting genes.^104^ Studies have also shown a strong interaction between SPP1+ TAMs and TECs, with spatial transcriptomics revealing the colocalisation of APLN+ TipSI cells with SPP1+ TAMs.^67^ In conclusion, the polarisation state and subtype diversity of macrophages in the TME determine their complex effects on angiogenesis (Figure 5a).

### 5.2 CAFs

The tumour stroma comprises various cell subtypes, with fibroblasts and ECs being among the most abundant. During tissue injury, these two cell types coordinate their activities to promote the regeneration of tissue. In cancer, however, such regenerative pathway is usurped by malignant cells that manipulate stromal cell interactions to facilitate tumour progression.^106^ Within the TME, CAFs support angiogenesis by secreting large quantities of VEGF‐A and other proangiogenic factors. Additionally, CAFs release platelet‐derived growth factor (PDGF‐C), which not only enhances EC resistance to anti‐VEGF therapies but also promotes further angiogenesis by inducing the release of other proangiogenic growth factors.^106^, ^107^, ^108^ PDGF‐C‐induced FGF2 activates ECs, fostering tumour growth and invasion while linking different types of tumour parenchyma and stroma.^108^ In addition to inducing VEGF expression, OPN also recruits proangiogenic monocytes into the TME.^107^ In metastatic breast and prostate cancers, TCs facilitate angiogenesis at metastatic sites by downregulating CAF‐derived thrombospondin‐1.^109^ Moreover, CAFs release enzymes and MMPs that degrade and restructure the ECM, establishing a supportive ECM for EC migration and neovascularisation. CAFs can also indirectly influence ECs and angiogenesis by regulating the levels of lactate and other metabolites within the TME (Figure 5b).

### 5.3 MDSCs

The immunosuppressive characteristics of MDSCs are essential in the TME.^110^ MDSCs are divided into two major subtypes: Monocytic MDSCs (M‐MDSCs) resemble monocytes, while polymorphonuclear MDSCs (PMN‐MDSCs) resemble neutrophils, both in terms of their phenotype and physical characteristics.^111^ Within the TME, MDSCs exhibit low phagocytic activity but promote angiogenesis, tumour invasion and immune tolerance via secreting nitric oxide, ROS and anti‐inflammatory cytokines.^112^ MDSCs facilitate neovascularisation through two primary mechanisms: by releasing growth factors such as bFGF VEGF, PDGF and prokineticin 2 (Bv8) and by remodelling the ECM through MMPs while reprogramming other cells towards a protumour phenotype.^113^, ^114^, ^115^, ^116^ As a protease, MMP9 promotes EC migration and accelerates angiogenesis. Besides promoting angiogenesis, VEGF possesses immunosuppressive functions, increasing the recruitment of Tregs and MDSCs.^114^ Furthermore, MDSCs can mimic TCs by promoting angiogenesis through exosomes containing proangiogenic factors such as VEGF‐A and miRNA‐126α.^117^, ^118^, ^119^ The angiogenic activity of MDSCs is driven primarily by the VEGF/JAK/STAT signalling pathway. Although direct interactions between ECs and MDSCs have not yet been explicitly described, their roles within the TME suggest a potential reciprocal influence, which future research may elucidate^114^, ^115^, ^120^, ^121^ (Figure 5c).

### 5.4 NK cells

Natural killer (NK) cells perform essential immunomodulatory functions within the TME.^122^ TECs in the TME can activate NKG2D by expressing RAE‐1ε, resulting in the internalisation of NKG2D from the surface of NK cells and the conveyance of inhibitory signals, resulting in NK cell dysfunction.^123^ Conversely, in poliomyelitis, NK cells activated by IL‐15 can proficiently eliminate ECs that have elevated expression of the nectin cell adhesion molecule 2 and poliovirus receptor.^124^ Additionally, cytokines including IL‐1β and TNF‐α, which are released by CD11b+ cells, can stimulate ECs to produce CCL7 and CCL2, thereby recruiting NK cells. This procedure additionally promotes the generation of VCAM‐1 and ICAM‐1, which facilitates stable contacts between ECs and NK cells^125^ (Figure 5d).

### 5.5 PCs

In the TME, abnormal interactions between pericytes and ECs can lead to tumour vascular malformation, neogenesis and dysfunction.^126^ After the formation of vascular sprouts, ECs secrete hepatocyte growth factor (HB‐EGF) and TGFβ1/2 to promote pericyte proliferation and recruitment.^127^ Subsequently, TCs activate ECs to produce Platelet‐Derived Growth Factor Subunit B (PDGF‐BB), which recruits PCs expressing Platelet‐Derived Growth Factor Receptor Beta (PDGFR‐β). These PCs, in turn, release Ang‐1 and VEGF‐A to promote EC sprouting and neovascularisation. Moreover, ECs secrete ANGPT1 and ANGPT2, which effectively bind to the TIE2‐R, disrupting the adhesion complex between PCs and ECs and thereby regulating angiogenesis within tumours.^128^ The PDGF‐BB/PDGFR‐β signalling pathway is crucial to this process.^129^ Studies have shown that inhibiting the expression of PDGFB reduces the recruitment of pericytes and impairs the integrity of blood vessels, thereby accelerating the process of tumour metastasis.^130^ Additionally, NG2 expressed by pericytes stabilises the contact between ECs and recruited PCs via a β1 integrin‐dependent manner and participates in pericyte recruitment and vascular maturation during angiogenesis by promoting collagen deposition.^131^ Conversely, PDGF‐BB derived from tumours can further induce ECs to attract PCs through the SDF‐1α/CXCR4 axis and increase the barrier coverage of tumour vessels.^132^ The high barrier coverage of PCs further protects TCs from drug attack and immune cell surveillance, correlating with unfavourable clinical prognosis.^133^ Notably, however, the barrier function of PCs also reduces the risk of tumour invasion^134^ (Figure 5e).

### 5.6 Neutrophils

Neutrophils, the predominant granulocytes in the bloodstream, are essential in promoting angiogenesis throughout the initial phases of cancer development, with the generation and expansion regulated by CSF3 (G‐CSF).^135^, ^136^ CXCL chemokines guide neutrophil migration to tumours through the CXCR1 and CXCR2 receptors.^137^, ^138^ Similar to TAMs, neutrophils constitute a significant source of proteases and proangiogenic factors. In mouse models, STAT3 signalling activates the transcription of VEGF‐A, FGF2 and MMP9, regulating the angiogenic functions of myeloid cells, including neutrophils.^139^ However, IFN signalling can inhibit STAT3, limiting neutrophil production of VEGF‐A and MMP9, thereby suppressing their angiogenic capabilities. Research has shown that IFN‐deficient mice exhibit accelerated tumour vascularisation and increased neutrophil infiltration than wild‐type mice.^137^, ^140^, ^141^, ^142^ Human neutrophils can release substantial amounts of VEGF under TNF stimulation.^142^ CSF3 enhances EC proliferation and angiogenesis by activating STAT3 to upregulate BV8 expression.^143^, ^144^ Consequently, blocking CSF3, CSF3R or BV8 can reduce neutrophil numbers and, in turn, inhibit angiogenesis.^135^ Additionally, activated neutrophils secrete proMMP‐9 devoid of tissue inhibitors of metalloproteinases, which induces angiogenesis by releasing fibroblast growth factor 2 (FGF‐2) and VEGF from the ECM. Neutrophils contribute significantly more to pro‐MMP9 production than TAMs^145^, ^146^ (Figure 5f).

### 5.7 Mast cells

Mast cells (MCs), as innate immune cells, influence the TME by modulating TC proliferation, angiogenesis, invasion and metastasis.^147^ MCs produce and secrete angiogenic factors including FGF and TGF‐β, along with chymase and tryptase, which influence ECs and TCs to promote their proliferation and metastasis.^148^ Several angiogenesis inhibitors that target tryptase, such as gabexate mesilate, may become new targets in future cancer treatments.^149^ The proangiogenic functions of MCs have been demonstrated in multiple malignancies, including skin cancer and gastric cancer.^150^, ^151^, ^152^, ^153^, ^154^ Furthermore, a favourable association exists between microvessel density and MC density^150^, ^154^ (Figure 5g).

### 5.8 T cells

In the TME, T cells influence tumour development and treatment by modulating immune responses. They can directly recognise and attack TCs or interact with other cells to collectively regulate the immune status.^155^ Studies indicate that TECs lacking PD‐L1 show a reduced ability to induce T‐cell apoptosis, ultimately suppressing tumour growth in vivo models.^156^, ^157^ Additionally, APLN+ TipSI cells induce T‐cell dysfunction and promote Treg infiltration, forming an immune‐suppressive microenvironment that supports angiogenesis. T cells can affect tumour growth via regulating angiogenesis. CD4+ Th1 cells secrete IFN‐γ, which inhibits EC proliferation and hinders angiogenesis^158^; CD4+ Th2 cells promote angiogenesis indirectly by secreting IL‐4, which activates TAMs.^159^ CD8+ cytotoxic T lymphocytes (CTLs) secrete IFN‐γ to enhance the expression of angiogenesis inhibitors in TAMs and use cytotoxic actions to kill tumours and ECs, disrupting the vascular network.^160^, ^161^ Conversely, Treg cells may promote angiogenesis through a variety of mechanisms, such as suppressing the overconsumption of VRGFA‐expressing Tregs and inhibiting IFN‐γ‐expressing T cells. Tregs can also inhibit other immune cells, such as CTLs, through cell contact‐dependent mechanisms, indirectly promoting tumour angiogenesis.^162^ Overall, the connections between T cells and ECs coregulate tumour growth and metastasis, highlighting their complex roles in tumour immunity (Figure 5h).

### 5.9 B cells

B cells are essential adaptive immune cells and are crucial in antitumour immunity.^163^ By releasing proangiogenic factors, including MMP9 and VEGF‐A, B cells facilitate tumour angiogenesis and support tumour invasion and metastasis.^164^, ^165^ Additionally, B cells activate cytokines through antigen‒antibody complexes, further enhancing TEC‐mediated angiogenesis.^166^, ^167^, ^168^ However, IgG1 antibodies can inhibit angiogenesis via the FcγRI pathway.^169^ Tumour‐secreted chemokines also regulate B‐cell activity, impacting angiogenesis.^170^, ^171^ The STAT3 signalling pathway is crucial in B‐cell‐mediated angiogenesis, with the HMGB1 protein in esophageal squamous cell carcinoma enhancing angiogenesis via altering the B‐cell signalling balance.^164^, ^172^, ^173^, ^174^ In chronic lymphocytic leukaemia, B cells abnormally express CD40L, causing ECs to secrete the B‐cell activation factors B‐cell‐activating factor and APRIL^175^ (Figure 5i).

## 6 ADVANCES AND EMERGING STRATEGIES IN AAT

### 6.1 Strategies targeting angiogenesis

Angiogenesis, a fundamental hallmark of cancer growth and survival, is a crucial factor for tumour growth and metastasis. Currently, various strategies targeting angiogenesis within the TME have been formulated, including methods to suppress angiogenesis, disrupt vascular, normalise the vasculature and promote angiogenesis (Table 1). By combining these diverse therapeutic strategies, more comprehensive treatments for tumours may be achieved.^176^ Here, we primarily discuss these four distinctive strategies that target angiogenesis.

**TABLE 1:** Current clinical development status of potential angiogenesis inhibitors.

Targets	Drug name	Alone or combination	Status	Phase	Condition	Identifier
FGFR	Bemarituzumab	Alone	Recruiting	I/II	Solid tumours	NCT05325866
		Chemotherap	Recruiting	III	GC/GEJA	NCT05052801
		CAPOX + SOX + nivoluma	Recruiting	I	GC/GEJ cancer	NCT05322577
	Rogaratinib	Combination drug	Completed	II	Cancer	NCT04125693
		Chemotherapy	Completed	II/III	UC	NCT03410693
		Alone	Completed	I	Cancer	NCT03788603
		Alone	Active, not recruiting	II	Advanced GIST	NCT04595747
		Alone	Completed	I	Cancer	NCT01976741
		Copanlisib	Completed	I	Solid tumours	NCT03517956
		Palbociclib + fulvestrant	Recruiting	I	BC	NCT04483505
		Atezolizumab	Active, not recruiting	I	UC	NCT03473756
	Erdafitinib	Cetrelimab	Recruiting	II	Bladder cancer	NCT06511648
		Alone	Active, not recruiting	II	Solid tumours	NCT06351371
		Alone	Recruiting	III	Bladder cancer	NCT06319820
		Alone	Active, not recruiting	II	Lymphoma	NCT06308822
		Alone	Recruiting	II	Brain cancer	NCT05859334
	Pemigatinib	Durvalumab	Not yet recruiting	II	BTC	NCT06530823
		Retifanlimab	Not yet recruiting	II	Liposarcoma	NCT06389799
		Afatinib	Recruiting	I	Solid tumour	NCT06302621
		Alone	Recruiting	II	Solid tumours	NCT06022289
		Alone	Recruiting	II	GIC	NCT05651672
		Alone	Recruiting	II	BC	NCT05559775
		Alone	Recruiting	II	NSCLC	NCT05287386
		Alone	Completed	II	NSCLC	NCT05253807
	Surufatinib	Same as VEGFR				
	Futibatinib	Alone	Not yet recruiting	II/III	Cancer	NCT06506955
		Pembrolizumab	Recruiting	II	Solid tumours	NCT05945823
		Alone	Not yet recruiting	II	CCA	NCT04189445
		Alone	Recruiting	II	CCA	NCT05727176
C‐Met	Tepotinib	Lazertinib	Not yet recruiting	II	NSCLC	NCT06106802
		Amivantamab	Recruiting	I/II	NSCLC	NCT06083857
		Alone	Not yet recruiting	II	NSCLC	NCT06031688
		Paclitaxel	Recruiting	I/II	GC/GEJ cancer	NCT05439993
		Pembrolizumab	Recruiting	I	NSCLC	NCT05782361
		Alone	Completed	I	HI	NCT03546608
	MCLA‐129	Same as EGFR				
	BYON3521	Alone	Active, not recruiting	I	Solid tumours	NCT05323045
C‐kit	Olverembatinib (HQP 1351)	Alone	Recruiting	II	MDS	NCT05521204
		Alone	Recruiting	III	CML	NCT05311943
		Decitabine	Recruiting	II	CML	NCT05376852
		Chemotherapy + sequential CAR‐T cells	Not yet recruiting	‒	Ph + ALL	NCT06481228
		Alone	Recruiting	III	CP‐CML	NCT06423911
		Decitabine + lisaftoclax	Not yet recruiting	I	CM/Ph + AML	NCT06401603
		Azacitidine + Bcl‐2 inhibitor	Not yet recruiting	‒	CML‐MBP	NCT06390306
VEGFR	BAT‐5906	Alone	Not yet recruiting	III	WAMD	NCT05439629
		Alone	Completed	II	WAMD	NCT05141994
		Alone	Completed	I/II	DME	NCT04772105
		Alone	Completed	I	WAMD	NCT04151212
	JY‐025	Alone	Unknown status	II/III	NSCLC	NCT04874844
	Olinvacimab	Pembrolizumab	Recruiting	II	TNBC	NCT04986852
	Ak109	AK104	Recruiting	III	GA/GEJA	NCT06341335
		AK104	Recruiting	I/II	Solid tumours	NCT05142423
		Paclitaxe	Recruiting	I/II	GA/GEJA	NCT04982276
		Alone	Completed	I	Solid tumour	NCT04547205
	CTX‐009	Alone	Active, not recruiting	II	CRC	NCT05513742
		Paclitaxel	Recruiting	II/III	BTC	NCT05506943
		Paclitaxel + irinotecan	Active, not recruiting	I/II	Solid tumours	NCT04492033
	9MW‐0813	Alone	Unknown status	III	DME	NCT05324774
		Alone	Completed	I	DME	NCT05324592
	Surufatinib	Immunotherapy	Recruiting	II	CRC	NCT05372198
		Alone	Recruiting	II	HCC	NCT05171439
		Fulvestrant + chidamide	Recruiting	II	BC	NCTO5186545
		Oxaliplatin + chemotherapy	Not yet recruiting	II	GC/GEJ cancer	NCT06447636
		Serplulimab + chemotherapy	Not yet recruiting	II	ESCC/BTC/GC	NCT06531291
		AG/FOLFIRINOX	Completed	I/II	PC	NCT05494580
		Nab‐paclitaxel + gemcitabine + camrelizumab	Recruiting	II/III	PC	NCT06361888
		Pamiparib	Recruiting	I/II	OC	NCT05494580
		Alone	Completed	I	Tumours	NCT02133157
		KN 046 + gemcitabine + nab‐paclitaxel	Recruiting	I/II	PC	NCT05832892
		Gemcitabine + nab‐paclitaxel	Not yet recruiting	II	PC	NCT06361030
		Camrelizumab + MFOLFOX6	PC		PC	NCT06329947
		Adebrelimab	Not yet recruiting	II	IHCC	NCT06375642
		Paclitaxel	Recruiting	II	OC	NCT06437353
		Toripalimab	Not yet recruiting	‒	IHCC	NCT06313554
	Telatinib	Same as PDGFR				
HGFR	YYB‐101	Alone	Completed	I/II	CRC	NCT04368507
		Alone	Completed	I	Solid tumours	NCT02499224
EGFR	MCLA‐129	Befotertinib	Not yet recruiting	I	NSCLC	NCT06015568
		Alone	Recruiting	I/II	Solid tumours/NSCLC	NCT04930432
		Alone	Recruiting	I/II	GC/NSCLC	NCT04868877
IGF‐1	VRDN‐001	Alone	Recruiting	III	TED	NCT06384547
		Alone	Recruiting	III	TED	NCT06179875
		Alone	Recruiting	III	TED	NCT06021054
		Alone	Active, not recruiting	III	TED	NCT05176639
	IGV‐001	Alone	Not yet recruiting	II	GBM	NCT04485949
PDGFR	Telatinib	Capecitabine + oxaliplatin	Unknown status	II	GC	NCT03817411
		Keytruda	Active, not recruiting	II	GC or HCC	NCT04798781
	Avapritinib	Decitabine	Recruiting	I	SM‐AHN	NCT06327685
		Azacitidine + decitabine	Recruiting	II	CBF‐AML	NCT06316960
		Alone	Recruiting	II	CBF‐AML	NCT05821738
		Alone	Active, not recruiting	/	GIST	NCT05381753

Abbreviations: ALL, acute lymphoblastic leukemia; BC, breast cancer; BTC, biliary tract cancer; CBF‐AML, core binding factor acute myeloid leukaemia; CCA, cholangio carcinoma; CML, chronic myeloid leukaemia; CML‐MBP, myeloid blast phase chronic myeloid leukaemia; CP‐CML, chronic phase chronic myeloid leukaemia; CRC, colorectal cancer; DME, diabetic macular oedema; EGFR, Epidermal Growth Factor Receptor; ESCC, esophageal squamous cell carcinoma; GA, Gastric Adenocarcinoma; GBM, glioblastoma; GC, gastric cancer; GIC, gastrointestinal cancer; GEJ, gastroesophageal junction; GEJA, gastroesophageal junction adenocarcinoma; GIST, gastrointestinal stromal tumour; HCC, hepatocellular carcinoma; HI, hepatic impairment; IHCC, intrahepatic cholangiocarcinoma; MDS, myelodysplastic syndromes; NSCLC, non‐small cell lung cancer; OC, ovarian cancer; PC, pancreatic cancer; PDGFR, platelet‐derived growth factor receptor; SM‐AHN, systemic mastocytosis with an associated haematologic neoplasm; TED, thyroid eye disease; TNBC, triple‐negative breast cancer; UC, urothelial carcinoma; VEGFR, vascular endothelial growth factor rgeceptor; WAMD, wet age‐related macular degeneration.

#### 6.1.1 Antiangiogenesis

Since Judah Folkman proposed in 1971 that ‘tumours depend on angiogenesis and angiogenesis inhibitors can treat cancer’, AATs have emerged as promising approaches.^37^ Bevacizumab, the inaugural angiogenesis inhibitor sanctioned by the FDA, is used in various cancers, including metastatic colorectal cancer and non‐small cell lung cancer (NSCLC). Although antiangiogenic treatment has shown significant effects, such as tumour shrinkage and prolonged survival, and has offered new hope to patients, it still faces substantial limitations, including limited efficacy, a lack of effective biomarkers and resistance. These challenges remain obstacles for AATs.^177^ In spontaneous orthotopic mouse cancer models, antiangiogenic agents fail to achieve sustained cancer control and, in some cases, even lead to increased cancer invasiveness or metastasis.

#### 6.1.2 Vascular normalisation

Vascular normalisation is a highly promising method for enhancing antitumour efficacy, aiming at reestablishing the integrity of the vascular system and improving drug delivery efficiency and antitumour immunity.^178^, ^179^ Compared with bevacizumab, ECs are more sensitive to low doses of PFKFB3 and CTAP inhibitors, effectively normalising the tumour vascular system and reducing metastasis.^180^, ^181^ Furthermore, research has shown that targeting vascular‐disrupting factors such as PAK4, LRG1 and PHGDH can effectively remodel the vascular microenvironment and increase the sensitivity to CAR‐T‐cell immunotherapy.^38^, ^39^, ^40^, ^41^, ^42^, ^43^, ^44^, ^45^, ^46^ Additionally, chloroquine has been shown to exhibit vascular normalisation effects independent of autophagy, which are associated with Notch signalling.^185^ Recent findings revealed that high‐dose vitamin C induces tumour vascular normalisation through TET2 activation.^185^ However, the effect of vascular normalisation is transient and requires precise dose control and timing of treatment to avoid overtreatment, which could lead to vascular damage and further tumour aggravation. Currently, reliable biomarkers to predict the therapeutic window are lacking.

#### 6.1.3 Vascular disruption

Vascular disruption strategies aim to directly damage or sever the tumour vascular network, depriving it of nutritional support and resulting in TC ischaemia and necrosis. Commonly used vascular‐disrupting agents (VDAs), such as the microtubule‐binding agent combretastatin, disrupt the cytoskeleton of TECs, whereas flavonoids exert toxic effects on vascular cells through proinflammatory effects, and ligand‐directed molecules deliver toxins or proapoptotic factors to TECs.^185^ In clinical trials, although VDAs have shown rapid tumour‐suppressing effects in cancer therapy, they have not substantially enhanced overall survival and progression‐free survival. VDAs pose cardiovascular toxicity risks and are often ineffective on the peripheral tumour vasculature, typically requiring a combination with chemotherapy or immunotherapy to increase treatment efficacy.^185^, ^186^ The application of VDAs in nanomedicine is also a direction worth exploring.^186^

#### 6.1.4 Proangiogenesis

Proangiogenesis strategies represent a relatively novel approach by enhancing the blood supply to tumours, thus improving drug delivery and oxygenation to increase therapeutic efficacy. Studies have shown that lysophosphatidic acid enhances drug delivery by specifically activating the LPA4 receptor in certain cancer models, ultimately promoting the development of vascular networks.^187^ The microtubule dynamics inhibitor eribulin mesylate, in combination with chemotherapeutic agents, has demonstrated proangiogenic effects and synergistic antitumour activity.^188^ Although these approaches have yet to be tested in humans and are not standard strategies in cancer treatment, they offer potential avenues for treating cancer by increasing vascular functionality.

### 6.2 Antiangiogenic immunotherapy

Antiangiogenic immunotherapy has become a prominent area of research in cancer treatment in recent years (Table 2). Its core principle lies in combining immune checkpoint inhibitors (ICIs) with antiangiogenic drugs to counteract the immunosuppressive state of the TME and promote vascular normalisation, thereby achieving synergistic antitumour effects.^189^, ^190^

**TABLE 2:** Ongoing clinical trials integrating antiangiogenic therapy with immunotherapeutic modalities.

NCT number	Study phase	Study status	Cancer type	Treatment
NCT06375486	II	Recruiting	Hepatocellular carcinoma	Ivonescimab (AK112, a PD‐1/VEGF bispecific antibody)
NCT05733611	II	Active, not recruiting	Colorectal cancer	Bevacizumab + atezolizumab + RP2/RP3
NCT04727307	II	Recruiting	HCC	Bevacizumab + atezolizumab
NCT04017455	II	Recruiting	Rectal cancer	Bevacizumab + atezolizumab
NCT04732598	III	Active, not recruiting	Breast cancer	Bevacizumab + atezolizumab + paclitaxel
NCT04721132	II	Recruiting	Hepatocellular carcinoma	Bevacizumab + atezolizumab
NCT01950390	II	Active, not recruiting	Melanoma (III‒IV)	Bevacizumab + ipilimumab
NCT04493203	II	Active, not recruiting	Melanom	Axitinib + nivolumab
NCT02853331	III	Active, not recruiting	Renal cell carcinoma	Axitinib + sunitinib + pembrolizumab
NCT04213170	II	Unkown status	Brain metastases	Bevacizumab + sintilimab
NCT04170556	I/II	Active, not recruiting	Hepatocellular carcinoma	Regorafenib + nivolumab
NCT03914300	II	Active, not recruiting	Thyroid gland carcinoma	Cabozantinib + nivolumab + ipilimumab
NCT06364631	III	Recruiting	Metastatic kidney cancer	Axitinib + nivolumab
NCT04443309	I/II	Recruiting	Hepatocellular carcinoma	Lenvatinib + camrelizumab (PD‐1 monoclonal antibody)
NCT04356729	II	Recruiting	Melanoma (III‒IV)	Bevacizumab + atezolizumab
NCT04749394	II	Recruiting	Non‐small cell lung cancer	Apatinib + camrelizumab
NCT04919629	II	Recruiting	Fallopian tube carcinoma	Bevacizumab + pembrolizumab + pegcetacoplan
NCT04981509	II	Recruiting	Renal cell carcinoma	Bevacizumab + erlotinib + atezolizumab
NCT06537908	‒	Recruiting	Hepatocellular carcinoma	Bevacizumab + IBI305 + anti‐PD‐1/PD‐L1 antibodies
NCT04715633	II	Active, not recruiting	Colorectal cancer	Apatinib + camrelizumab
NCT05332496	‒	Recruiting	Hepatocellular carcinoma	PD‐1/PD‐L1 inhibitors + VEGF‐TKI/bevacizumab + TACE
NCT06529718	II	Not yet recruiting	Biliary tract cancer	Ivonescimab + FOLFOX
NCT06530251	I/II	Not yet recruiting	Hepatocellular carcinoma	AK112 (PD‐1/VEGF bispecific antibody)
NCT06472895	I/II	Not yet recruiting	Renal cell carcinoma	AK112
NCT06402435	II	Not yet recruiting	Breast cancer	SBRT + AK112 + chemotherapy
NCT06400160	I/II	Not yet recruiting	Prostate cancer, colorectal cancer	Pembrolizumab + TB511
NCT06339424	II	Not yet recruiting	Hepatocellular carcinoma	Bevacizumab + atezolizumab + photon radiotherapy
NCT06375486	II	Recruiting	Hepatocellular carcinoma	Vonescimab + HAIC
NCT06374602	II	Recruiting	Anaplastic thyroid cancer	Lenvatinib + pembrolizumab
NCT06370065	II	Recruiting	Hepatocellular carcinoma	HLX04 (VEGF antibody) + HLX10 (PD‐1 antibody) + HAIC
NCT06232564	II	Recruiting	Neuroendocrine tumours	Pembrolizumab + carboplatin + etoposide + lenvatinib
NCT06006923	II	Recruiting	MSI‐H colorectal cancer	Regorafenib + pembrolizumab
NCT06196775	II	Not yet recruiting	Hepatocellular carcinoma	AK112 + cadonilimab (anti‐PD‐1/CTLA‐4)

*Note*: Status according to https://clinicaltrials.gov/, accessed on 6 August 2024. Immunotherapy drugs—anti‐PD‐1: pembrolizumab, nivolumab, sintilimab, ivonescimab and AK112; anti‐PD‐L1: atezolizumab, durvalumab and avelumab; anti‐CTL4: ipilimumab. Antiangiogenic therapies—anti‐VEGF: bevacizumab, IBI305, ivonescimab and AK112; anti‐VEGFRs: apatinib, axitinib, sunitinib, regorafenib, cabozantinib and lenvatinib; chemotherapy: paclitaxel, carboplatin and etoposide.

Abbreviations: SBRT, stereotactic radiotherapy; TACE, transarterial chemoembolisation.

Vascular abnormalities contribute to the immune evasion of tumours, while the use of antiangiogenic drugs to normalise the vasculature simultaneously opens the door for T‐cell infiltration into tumours. Preclinical evidence suggests that antiangiogenic immunotherapy has significant potential for enhancing cancer treatment outcomes.^191^, ^192^, ^193^, ^194^, ^195^, ^196^, ^197^, ^198^ The Ang2 and TIE pathways are critical therapeutic targets in antiangiogenic immunotherapy.^191^, ^192^, ^193^ Studies have shown that conditional deletion of Tie1 reduces microvascular density, improves vascular normalisation, and increases intratumoural necrosis. In transgenic and transplanted tumour models, the blockade of VEGF‐A and ANGPT2 by bispecific antibody (A2V) significantly enhances antitumour immunity and promotes the accumulation of CD8+ CTLs.^194^ On the other hand, the modulation of tumour lymphatics and the induction of tertiary lymphoid structures via LIGHT–VTP substantially augmented the quantity of memory T cells and intratumoural effector T cells, increasing survival rates.^195^ Additionally, research has shown that combined anti‐PD‐L1 and anti‐VEGFR2 antibody therapy induced high levels of endothelial venules in mouse models, stimulating tumour immunity.^196^ Yang's research indicates that STING regulates tumour blood vessels and has synergistic effects with ICIs and anti‐VEGFR2 agents.^197^ Interestingly, ivonescimab (A2V blockade of VEGF and PD‐1) monotherapy showed significant effectiveness in patients with metastatic NSCLC.^198^ Clinically, research has demonstrated that combined VEGF2 and PD‐1 inhibitor therapy provides greater clinical benefits in HCC and RCC than monotherapy does.^199^, ^200^, ^201^, ^202^, ^203^ In treating NSCLC and RCC, bevacizumab in combination with immunotherapy has demonstrated superior survival advantages over single‐agent therapy. Recently, Deng et al. proposed a novel method using nanoparticles of gambogenic acid for the combined application of antiangiogenic and immunotherapeutic strategies.^204^ Nonetheless, numerous obstacles remain in optimising the therapeutic efficacy of these combination therapies. First, the current lack of robust predictive biomarkers hinders the precise identification of patients, necessitating urgent efforts to identify predictive biomarkers for screening potential beneficiaries. Second, determining the optimal combination regimen is critical. This includes shifting the focus from conventional anti‐VEGF/R agents to other candidates (e.g., FGF/R inhibitors) and evaluating whether the combination exerts synergistic rather than only additive effects. Third, the organ‐specific heterogeneity of angiogenic phenotypes complicates therapeutic interventions, as a universal approach is unlikely to suit all tumour types. Thus, in‐depth analyses are required to develop organ‐specific personalised strategies. Finally, variations in treatment sequencing and dosing intervals may significantly impact outcomes, underscoring the need to explore optimal therapeutic schedules.^205^

### 6.3 Application of nanodrug delivery systems

In recent years, the application of nanotechnology in AAT has demonstrated immense potential, with tumour‐targeted nanodrug delivery systems gaining significant attention in cancer treatment because of their tumour specificity, efficient drug delivery and resistance to drug resistance mechanisms.^206^

Targeted drug delivery can be classified into ‘passive’ and ‘active’ types.^207^ ‘Passive’ targeting utilises the unique features of the tumour vasculature, improving nanodrug delivery efficiency through enhanced permeability and retention effects, which has shown promising results in preclinical models.^208^ Increased tumour vascular permeability is a key factor in the enhanced permeability and retention effect and is closely related to the degree of vascularisation. Studies have shown that, in lung injuries, nanocarriers can interact with the pulmonary endothelium via passive targeting to restore homeostasis, demonstrating considerable therapeutic activity.^209^ Additionally, inorganic nanoparticles can induce endothelial leakage, enhancing the ability of drugs to cross vascular barriers.^210^ ‘Active’ targeting, on the other hand, involves ligand modifications on the nanocarriers, allowing their binding to particular sites and improving the accuracy of drug delivery.^211^

Combining angiogenesis inhibitors or VDAs with nanodrug technology to modulate tumour vascular normalisation has demonstrated efficacy as an approach for improving cancer therapy.^212^, ^213^ Chauhan et al. found in breast tumour trials that inhibiting VEGFR‐2 could ‘normalise’ the tumour vasculature, enhancing the administration of tiny nanodrugs, while larger nanoparticles have difficulty penetrating the tumour.^214^ Jiang et al. reported that restoring tumour vasculature enhanced the administration of medium‐sized nanoparticles up to 40 nm in size.^215^ Cun et al. developed multifunctional size‐switchable nanoparticles that showed promising effects in vascular normalisation.^216^ Additionally, inhibiting the Nogo‐B receptor (NgBR) can reduce EC migration, thereby suppressing angiogenesis.^217^ Jiang et al. designed jet‐lagged nanoparticles combined with vascular normalisation and metabolic therapy using apatinib‐loaded nanomedicines to block VEGF/VEGFR2 and effectively inhibit tumour vascular proliferation.^218^ Interestingly, a DNA nanorobot loaded with thrombin and specifically delivered to the tumour vasculature can trigger intratumoural thrombosis, inducing vascular infarction and ultimately leading to tumour necrosis.^213^ Nanodrug‐based strategies demonstrate substantial potential in cancer therapy; however, they confront multiple challenges in drug release control, biocompatibility, safety evaluation and cost‐effectiveness.^219^ First, imprecise control over drug release often leads to off‐target accumulation, elevating the risk of systemic toxicity. Second, biocompatibility challenges arise from the suboptimal biodistribution and metabolism of nanoparticles, which are swiftly eliminated by the mononuclear phagocyte system. This results in an excessive buildup in organs such as the spleen and liver, compromises drug availability at tumour sites, and triggers immune activation. Furthermore, the prolonged in vivo accumulation as well as the possible toxicity of nanoparticles remain incompletely characterised, posing safety concerns for clinical translation. Finally, the complex fabrication processes and high production costs associated with nanodrugs—involving diverse materials and intricate techniques—hinder large‐scale manufacturing and broad clinical implementation. Addressing these critical barriers through innovative research is critical to realising the full therapeutic promise of nanomedicine in oncology.^220^

### 6.4 Other promising targeting strategies

Antiangiogenic strategies have also been explored for methods to influence tumour metabolism and disrupt the TME. The metabolism of ECs, as a new and promising target within the TME, is becoming a hot research direction in clinic.^221^ Recently, Fang et al. demonstrated that focusing on fructose metabolism could serve as an effective strategies to diminish angiogenesis and inhibit tumour proliferation. Furthermore, targeting PFKFB3 or CPT1a has been shown to result in a reduction of pathological angiogenesis as well as the normalisation of tumour vasculature, signifying a prospective novel therapeutic target for cancer therapy.^13^ Angiocrine factors are associated with multiple facets of cancer progression, including proliferation, stemness, invasion, epithelial‐mesenchymal transition and immunosuppression. For instance, studies have demonstrated that focal adhesion kinase in ECs acts as a pivotal regulator of chemotherapy sensitivity. Inhibition of FAK expression disrupts EC‐derived paracrine signals (also termed angiocrine signals), thereby suppressing tumour growth.^222^, ^223^ EndMT in ECs can result in organ fibrosis and cancer development, involving the regulation of cytoskeletal components.^70^, ^224^ A key strategy for targeting EndMT involves the development of antibody‒drug conjugates that inhibit microtubule protein polymerisation and target TGF‐β signalling, thereby delaying the progression of diseases such as tumours.^225^, ^226^, ^227^, ^228^, ^229^, ^230^, ^231^ In the past few years, with the growing volume of research on tumour‐derived exosomes (TDEs) targeting ECs to promote tumour metastasis, the strategy of targeting TDEs has shown broad prospects in inhibiting tumour metastasis. Olejarz et al. explored the application of exosomes in angiogenesis and suggested that controlling the exosomal composition could be a promising method to increase the long‐term effectiveness of AAT.^232^ Currently, it has been found that cannabidiol, Chloramidine/bisindolylmaleimide‐I, and traditional Chinese medicine can inhibit the release of TDEs to impede cancer progression. However, the targeted delivery of TDEs remains a challenge.^233^, ^234^, ^235^, ^236^ Recently, strategies aimed at altering vascular phenotypes to optimise cancer treatment have gained rapid momentum, primarily related to the induction of high endothelial venules within tumours, which promote the extravasation of lymphocytes and correlate with improved prognoses in multiple cancers. In fibrosarcoma models, the absence of Tregs results in the emergence of high endothelial venules, enabling T‐cell recruitment.^176^, ^237^ The glycocalyx (GCX) is essential for the interaction between ECs and TCs. Targeting endothelial GCX can inhibit tumour metastasis by regulating vascular permeability. Study shows that TCs enhance the establishment of an adhesive vascular niche by depositing GCX components along ECs, thereby facilitating their extravasation. Targeting the hyaluronic acid‐CD44 GCX complex significantly reduces the extravasation of TCs, providing new evidence for developing therapeutic strategies against metastasis.^238^ The exploration and development of novel targets are conducive to more precisely interfering with the tumour vasculature. Zhang et al. discovered that high expression of DGKG promotes angiogenesis as well as immune evasion in HCC, suggesting that targeting endothelial DGKG may be a viable approach for precision therapy.^239^ A deeper comprehension of the biological properties of tumour vasculature in the future will allow for more effective cancer interventions and the formulation of novel approaches to therapy.

### 6.5 Resistance to AAT and countermeasures

The mechanisms of resistance to AAT have historically been a considerable challenge in cancer treatment. These resistance mechanisms are complex and diverse, involving TCs, ECs and various factors within the TME. First, tumours escape treatment pressure by activating alternative angiogenic modes, such as IA, vasculogenic mimicry and vessel co‐option.^240^, ^241^, ^242^ For example, Saravanan et al. found that the IA mechanism can bypass VEGF inhibition to continuously drive tumour angiogenesis.^243^ Teuwen et al. revealed that anti‐VEGF treatment‐induced vessel co‐option depends on the synergistic action of invasive cancer cell subtypes and matrix‐remodelling macrophages.^244^ Cannell et al. confirmed that the Foxc2‐driven vasculogenic mimicry pathway is a key mechanism of resistance in gliomas and other solid tumours.^245^ Second, persistent vascular dysfunction prompts ECs to undergo metabolic reprogramming and activate compensatory signalling pathways, thereby significantly promoting drug resistance. After VEGF inhibition, ECs maintain survival through alternative signalling pathways such as Angiopoietin/Tie2, Notch, PDGF and FGF.^246^, ^247^, ^248^, ^249^, ^250^, ^251^ For example, the LRG1‒TGF‐β axis can activate the BMP9/ALK1 signalling pathway in ECs.^252^ In addition, during AAT, TCs undergo phenotypic changes. For example, the stimulation of the HGF/c‐MET pathway enhances tumour invasiveness. In patients with glioblastoma, the expression of c‐MET is significantly elevated after recurrence following bevacizumab therapy.^113^, ^253^ Meanwhile, tumour heterogeneity also leads to differences in sensitivity to antiangiogenic drugs. Finally, immune‐suppressive cells (e.g., Tregs and MDSCs) weaken the effects of AAT by forming immunosuppressive niches. These mechanisms collectively lead to resistance to AAT.

Breakthroughs in overcoming resistance to AAT will rely on the coordinated advancement of multiple strategies. In terms of biomarker development, blood flow and volume may serve as a potential biomarker. For example, Chen et al. successfully predicted differences in bevacizumab response among patients (colorectal cancer liver metastasis) by quantitatively assessing vessel co‐option and angiogenic features through liver CT scans, providing a model for the clinical application of imaging biomarkers.^254^ In endometrial cancer, TP53 mutations and p53 overexpression have been shown to be significantly associated with bevacizumab sensitivity.^255^ Regarding combination therapy, clinical and preclinical investigations have shown that the combination of antiangiogenic drugs with ICIs significantly enhances treatment efficacy, representing an important direction for future combination therapies. Moreover, precisely grasping the ‘therapeutic window’ is a hidden lever for improving efficacy: in a glioblastoma model, the CXCR4 inhibitor AMD3100 effectively inhibited angiogenesis only when administered early after radiotherapy to block SDF‐1‐mediated recruitment of vascular precursors, but was ineffective at later time points.^256^, ^257^ This indicates that in treatments aimed at preventing post‐treatment vascular regeneration and tumour recurrence, strict adherence to the therapeutic window is necessary to ensure effective intervention. In summary, These advances not only chart a path towards overcoming AAT resistance but also lay the groundwork for personalised therapeutic paradigms.

## 7 CONCLUSION AND FUTURE PERSPECTIVE

In summary, we have discussed the relationship between tumours and ECs within the TME, highlighting the pivotal function of angiogenesis in influencing tumour proliferation and metastasis. In addition, we have summarised the latest advancements and strategies in targeting angiogenesis. Although some studies have validated these findings in preclinical cancer models, their specific clinical application remains an unresolved issue. Future study objectives may encompass the formulation of novel pharmaceuticals and therapeutics to more precisely target the tumour vasculature. Another important research direction involves targeting the interplay between tumour vasculature and other elements of the TME, including immune cells. Furthermore, it is essential to explore how to integrate various therapeutic modalities with vascular‐targeted treatments to augment therapeutic efficacy.

On the other hand, in the context of therapeutic strategies targeting the TME, sophisticated technologies such as single‐cell sequencing and spatial transcriptome sequencing have revealed the complex heterogeneity of the tumour vasculature, which provides important clues for researchers to enhance their comprehension of cancer mechanisms and formulate novel treatment strategies. For instance, strategies can be designed to specifically target different EC subpopulations based on their heterogeneity. The pan‐cancer EC atlas constructed by Li et al. shows that the abundance of the CXCR4+ tip cell subpopulation is negatively correlated with sensitivity to AAT, while the absence of the SELE+ venous subpopulation may indicate a decreased response rate to immunotherapy, laying a theoretical foundation for the future advancement of AATs.^66^ In addition, single‐cell sequencing technology can also aid in the development of biomarkers to predict the response to AAT. For example, the APLN+ TipSI cells defined by Pan et al. can serve as a biomarker for dynamic assessment of bevacizumab efficacy, with a reduction in their infiltration post‐treatment being directly related to extended patient survival.^67^

As an emerging approach, AAT possesses the capacity to offer significant clinical advantages for patients with cancer and antitumour treatments. In the coming years, we assert that with an in‐depth comprehension of angiogenesis, the TME, and resistance mechanisms, the challenges associated with AATs may be addressed quickly.

## AUTHOR CONTRIBUTIONS

Zhen Li, Quanbo Zhou and Weipeng Sun conceived and supervised the study, provided critical intellectual content revisions and approved the final manuscript. Shuaixi Yang, Yingshuai Fang and Yangcheng Ma contributed equally to the conceptual framework and designed the methodology. Fuqi Wang, Yuhang Wang, Jiachi Jia and Yabing Yang conducted systematic literature curation and drafted the original manuscript with visualisation support. All authors participated in iterative revisions, interpreted findings and endorsed the submitted version.

## CONFLICT OF INTEREST STATEMENT

The authors declare they have no conflicts of interest.

## ETHICS STATEMENT

The authors have nothing to report.

## Data Availability

Data sharing is not applicable to this article as no new data were created or analysed in this study.
